# De Garengeot Hernia Presenting as a Rare Cause of Incarcerated Femoral Hernia

**DOI:** 10.7759/cureus.102498

**Published:** 2026-01-28

**Authors:** Michael W Alchaer, Harrison Gorran, Amanda Rigdon, Thomas A Abbruzzese, Ji Fan

**Affiliations:** 1 Medicine, University of South Florida Morsani College of Medicine, Brandon Regional Hospital, Brandon, USA; 2 General Surgery, University of South Florida Morsani College of Medicine, Brandon Regional Hospital, Brandon, USA; 3 Internal Medicine, Lakeland Regional Health Medical Center, Lakeland, USA; 4 Gastrointestinal Surgery, Moffitt Cancer Center, Tampa, USA

**Keywords:** appendicitis, de garengeot hernia, emergency surgery, femoral hernia, open repair

## Abstract

De Garengeot hernia is defined as the presence of the appendix within a femoral hernia sac and represents a rare surgical condition. Its clinical presentation closely resembles that of an incarcerated or strangulated femoral hernia, often leading to delayed or intraoperative diagnosis.

We report the case of an elderly woman who presented with acute right-groin pain and an irreducible groin mass concerning for an incarcerated femoral hernia. Emergent open exploration through an inguinal approach revealed a gangrenous appendix within the femoral canal. Appendectomy and primary tissue repair were performed, and the patient recovered uneventfully.

Due to nonspecific clinical findings, De Garengeot hernia is most frequently diagnosed intraoperatively. Computed tomography may assist in preoperative diagnosis when a blind-ended tubular structure contiguous with the cecum is identified within the femoral canal. Prompt surgical intervention is essential to prevent perforation and infectious complications. While laparoscopic repair may be considered in selected stable patients, open exploration remains the standard approach in emergency settings.

Maintaining a high index of suspicion for De Garengeot hernia in elderly women presenting with irreducible groin masses allows for timely diagnosis and definitive surgical management with excellent outcomes.

## Introduction

De Garengeot hernia, first described by René Jacques Croissant de Garengeot in 1731, refers to the rare presence of the vermiform appendix within a femoral hernia sac [1,2]. It represents approximately 0.5-1% of all femoral hernias and fewer than 0.1% of all appendicitis cases [2-4]. The condition occurs predominantly in elderly females due to a wider femoral canal and higher incidence of femoral herniation [1,2].

The pathogenesis is thought to involve cecal mobility or congenital elongation of the appendix that allows its descent through the femoral canal [2,5]. Inflammation usually results from compression and ischemia at the narrow hernial neck rather than primary appendicitis [2,5,6]. Because its presentation mimics that of a strangulated femoral hernia, diagnosis is generally intra-operative [1,2,5]. Immediate surgery is therefore recommended to avoid perforation or abscess formation [2,6].

Presented as a poster in the 2025 Surgical of American Gastrointestinal and Endoscopic Surgery (SAGES) Annual Meeting in Long Beach, California.

## Case presentation

An 89-year-old female patient presented with a one-day history of progressively worsening right groin pain and swelling. Her medical history was notable for endometrial cancer, severe aortic stenosis, and chronic atrial fibrillation, placing her in a high-risk surgical category. On examination, a firm, tender, non-reducible 6 cm bulge was palpated inferior to the right inguinal ligament, raising concern for a complicated femoral hernia. Initial laboratory evaluation demonstrated leukocytosis of 16 × 10³/µL, suggestive of an underlying inflammatory or infectious process. Given the constellation of findings, acute onset of pain, an irreducible groin mass, and leukocytosis, a diagnosis of strangulated femoral hernia was made, and the patient was taken emergently to the operating room.

**Figure 1:**
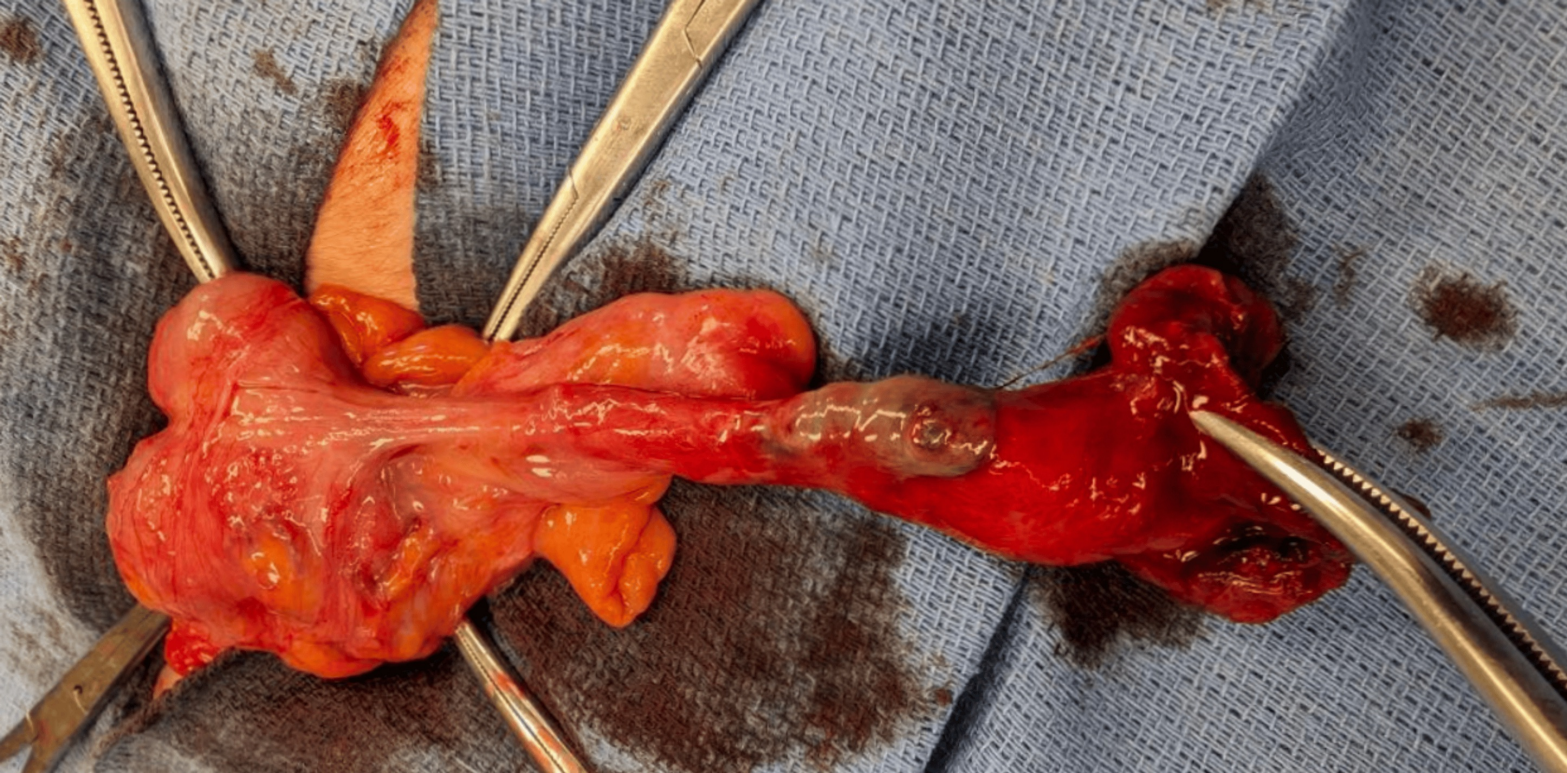
Intraoperative appendix post extraction

Intraoperatively, an incarcerated femoral hernia was confirmed. Upon opening the hernia sac, a gangrenous, necrotic appendix was unexpectedly identified, consistent with a De Garengeot hernia (Figure 1). The appendix appeared ischemic due to prolonged entrapment within the femoral canal. An appendectomy was performed, and the viable cecum was carefully reduced back into the peritoneal cavity. The femoral defect was repaired anatomically using the McVay (Cooper's ligament) herniorrhaphy technique. Given her advanced age, significant comorbidities, and the emergent nature of the operation, her postoperative course was complicated and overall not remarkable for rapid recovery.

## Discussion

De Garengeot hernia is a rare surgical curiosity in which the vermiform appendix lies within a femoral hernia sac [2]. It accounts for <1% of femoral hernias and <0.1% of all appendicitis cases, with <200 cases documented worldwide [2,4]. The entity predominantly affects elderly women, reflecting both the greater incidence of femoral hernias in this group and the wider femoral canal anatomy conferred by pelvic morphology [1,4,6]. Additional predisposing factors include chronic increases in intra-abdominal pressure, connective-tissue laxity, and cecal mobility that permit appendiceal descent through the femoral ring [1-3,5].

The pathogenesis of inflammation in De Garengeot hernia remains debated; the prevailing hypothesis is vascular compromise of the incarcerated appendix secondary to compression at the rigid femoral neck rather than primary luminal obstruction [2,5,6]. This ischemic mechanism explains why suppurative or gangrenous appendicitis within a femoral hernia is often an effect rather than a cause of incarceration [2,6-8]. Histologic studies corroborate this view, showing transmural necrosis and periserosal congestion without proximal fecaliths [2,5]. Other anatomic contributors include a long mesoappendix or mobile cecum that facilitates migration into the femoral canal [3,9].

Clinically, patients present with a painful, irreducible groin mass often mistaken for a strangulated femoral hernia [1,2]. Because the inflamed appendix is confined within a tight space, classical signs of appendicitis, migratory pain, fever, and leukocytosis, may be absent, leading to diagnostic ambiguity [2,10]. Although computed tomography can occasionally demonstrate a blind-ended, tubular structure within the femoral canal contiguous with the cecum, most De Garengeot hernias are diagnosed intra-operatively due to their rarity and the urgency of presentation, which often precludes extensive preoperative imaging [1,2]. Consequently, most cases are recognized intra-operatively. In the systematic review by Guenther et al., over 90% were diagnosed only at surgery [2].

Given the risk of ischemic necrosis, perforation, or abscess formation, prompt surgical exploration is imperative once suspected [2]. The optimal approach depends on patient factors and intra-operative findings [1,10]. Open femoral repair via an infra-inguinal incision remains standard when strangulation is suspected or bowel viability must be assessed [2]. Laparoscopic transabdominal preperitoneal (TAPP) or totally extraperitoneal (TEP) repair offers superior visualization, the ability to inspect contralateral hernias, and shorter recovery in stable patients [11].

The simultaneous occurrence of acute appendicitis within a femoral hernia complicates both the clinical picture and the operative strategy [1,2]. Attempting manual reduction in the emergency department is discouraged, as it can exacerbate ischemia or cause perforation of the entrapped appendix [2]. Surgical management remains debated: while some advocate a combined low midline and inguinal approach, others favor a single incision technique, which, when feasible, allows for both appendectomy and hernia repair through one exposure [2,11].

When recognized early and treated appropriately, outcomes are excellent [2,11]. Reported mortality approaches zero, and recurrence is exceedingly uncommon [2,12]. Delayed diagnosis, however, may lead to sepsis or femoral abscess formation [13-15]. Laparoscopic repair offers faster convalescence and lower wound infection rates compared with open techniques in suitable candidates [11,16,17].

## Conclusions

De Garengeot hernia remains a surgical rarity that underscores the importance of maintaining diagnostic vigilance when evaluating irreducible groin masses, particularly in elderly females. Because its presentation often mimics a strangulated femoral hernia, prompt operative exploration is essential for both diagnosis and treatment. Inflammation within the femoral canal is usually secondary to ischemic compromise rather than primary appendicitis, and early intervention prevents perforation, abscess formation, and sepsis. Although laparoscopic repair can be safely performed in stable patients and offers reduced wound complications, open repair remains the standard approach in emergencies. Awareness of this entity among surgeons and radiologists facilitates timely recognition and optimal outcomes, with mortality approaching zero and recurrence exceedingly rare.
